# Identification of Distinct Clinical Phenotypes of Critically Ill COVID-19 Patients: Results from a Cohort Observational Study

**DOI:** 10.3390/jcm12083035

**Published:** 2023-04-21

**Authors:** José Pedro Cidade, Vicente Cés de Souza Dantas, Alessandra de Figueiredo Thompson, Renata Carnevale Carneiro Chermont de Miranda, Rafaela Mamfrim, Henrique Caroli, Gabriela Escudini, Natalia Oliveira, Taiza Castro, Pedro Póvoa

**Affiliations:** 1 Intensive Care Unit 4, Department of Intensive Care São Francisco Xavier Hospital, CHLO, Lisbon, 1449-005 Lisbon, Portugal; 2 Nova Medical School, Clinical Medicine, CHRC, New University of Lisbon, 1169-056 Lisbon, Portugal; 3 Instituto D’Or de Pesquisa e Ensino, Rio de Janeiro 22281-100, Brazil; 4 Hospital Copa D´Or, Rio de Janeiro 22031-011, Brazil; 5 Center for Clinical Epidemiology, Research Unit of Clinical Epidemiology, OUH Odense University Hospital, 5000 Odense C, Denmark

**Keywords:** COVID-19, phenotypes, mortality rate, cluster analysis, critical care

## Abstract

**Purpose**: COVID-19 presents complex pathophysiology, and evidence collected points towards an intricate interaction between viral-dependent and individual immunological mechanisms. Identifying phenotypes through clinical and biological markers may provide a better understanding of the subjacent mechanisms and an early patient-tailored characterization of illness severity. **Methods:** A multicenter prospective cohort study was performed in 5 hospitals in Portugal and Brazil for one year between 2020–2021. All adult patients with an Intensive Care Unit admission with SARS-CoV-2 pneumonia were eligible. COVID-19 was diagnosed using clinical and radiologic criteria with a SARS-CoV-2 positive RT-PCR test. A two-step hierarchical cluster analysis was made using several class-defining variables. **Results:** 814 patients were included. The cluster analysis revealed a three-class model, allowing for the definition of three distinct COVID-19 phenotypes: 407 patients in phenotype A, 244 patients in phenotype B, and 163 patients in phenotype C. Patients included in phenotype A were significantly older, with higher baseline inflammatory biomarkers profile, and a significantly higher requirement of organ support and mortality rate. Phenotypes B and C demonstrated some overlapping clinical characteristics but different outcomes. Phenotype C patients presented a lower mortality rate, with consistently lower C-reactive protein, but higher procalcitonin and interleukin-6 serum levels, describing an immunological profile significantly different from phenotype B. **Conclusions:** Severe COVID-19 patients exhibit three different clinical phenotypes with distinct profiles and outcomes. Their identification could have an impact on patients’ care, justifying different therapy responses and inconsistencies identified across different randomized control trial results.

## 1. Introduction

The SARS-CoV-2 infection causes a wide spectrum of clinical manifestations, ranging from the complete absence of symptoms to a severe acute respiratory syndrome with a high fatality rate [1].

The natural history and pathophysiology of COVID-19 involves complex mechanisms, including viral infection and replication, immunological mechanisms, and a broad dysregulation of clotting, host defense, and endothelial dysfunction [2,3]. The evidence collected shows that many clinical and biochemical parameters are altered in COVID-19 patients, and this seems to be correlated with the severity of the disease and its prognosis [4].

On that regard, the characterization of clinical phenotypes within COVID-19 infected patients may represent a paramount clinical step, allowing us to increase our knowledge about the mechanisms of this disease and to comprehend inherent changes in patients’ diagnoses, prognoses, and treatment responses [5]. Azoulay et al. and Fish et al. have already provided insights into the biological plausibility of these phenotypes and their unique associations between several different biomarkers and patients’ prognoses [6,7]. Several clinical studies have further extended the characterization of these COVID-19 clinical phenotypes. Bruse et al. aimed to apply previously established clinical sepsis phenotypes to patients with COVID-19. The authors identified different phenotype distribution and biomarkers profiles when comparing to those previously established, confirming the need to conclusively establish clear clinical phenotypes to aid prognostication and prediction of treatment efficacy [8]. Siepel et al. attempted to confirm these clinical phenotypes using an unsupervised machine learning analysis. The authors conclude that the dynamic interchange of patients through phenotypes over time could explain the heterogeneity of results when aiming to describe these COVID-19 clinical phenotypes [9]. This evidence was in agreement with more recent studies that found similar clinical phenotypes across different countries [10,11,12,13,14,15,16].

The role of different biomarkers profiles in defining these COVID-19 phenotypes in critically ill patients has yet to be confirmed, but it could potentially aid to characterize these clinical phenotypes and to predict patients’ outcomes. In fact, biomarkers have been shown to be able to provide an assessment of several organ dysfunctions in COVID-19 patients, such as the risk of acute respiratory distress syndrome and disseminated intravascular coagulation [17,18]. Patients who present with lymphopenia, elevated D-dimer and troponin, hyperferritinemia, and increased lactic dehydrogenase usually have an unfavorable course of the disease [19]. Other non-specific biomarkers of COVID-19, such as the presence of neutrophilia, thrombocytopenia, hypoalbuminemia, elevation of liver enzymes and creatinine, as well as inflammatory markers, such as C-reactive protein (CRP) and interleukin 6 (IL-6), were also associated with a worse prognosis [20,21,22,23,24,25,26].

Blair et al. tried to stratify COVID-19 patients in different clusters using distinct blood inflammatory biomarkers. Their results pointed to three distinct inflammatory biomarker patterns that could stratify a heterogeneous population and that were associated with comorbid diseases and illness severity of those patients. This exploratory step gave important insights regarding the inclusion of biomarkers in the definition of clinical phenotypes, suggesting that these could be used for a personalized approach to the triage of care and therapeutic indications [27].

It has become progressively clearer that this biomarker phenotyping could lead the way to a more precise and personalized care for COVID-19 patients, highlighting the possibility of future treatments and interventions taking into account the disease’s particularities and patients’ predisposition and response [5,28,29]. Notably, the refinement of trials through prognostic enrichment is expected to increase the likelihood for beneficial effects of an intervention to emerge. Following these developments, this precision medicine paradigm may enable the tailoring of treatment to apparently similar patients with vastly different biochemical profiles and outcomes [30,31,32].

The main aim of our study is to assess and identify COVID-19 patients’ phenotypes using clinical and biomarker profiles, allowing the establishment of plausible correlations with clinical outcomes.

## 2. Materials and Methods

### 2.1. Design and Setting

This was a prospective observational cohort study performed in five ICUs of Brazil and Portugal. Briefly, during the study period (March 2020 to June 2021), we evaluated every adult patient (≥18 year) who required ICU admission with PCR confirmed SARS-CoV-2 infection.

### 2.2. Definitions, Selection of Participants and Data Collection

The Ethics Committees of the Hospital Copa Star and Hospital Copa D’or in Rio de Janeiro, Brazil, and of the Portuguese Ethics Committee for Clinical Investigation in Lisbon, Portugal, approved the study (CAAE: 17079119.7.0000.5249; and REC: 2020_EO_02, respectively) and we received the waiver of the written informed consent.

Sample size was calculated considering COVID-19’s population prevalence from Portugal and Brazil at the time of the study’s proposal, using the highest limit of the 95% confidence level and considering a 5% marginal error. We collected demographic, clinical, and laboratory data using standardized case report forms and patients’ daily chart revisions, and included the Simplified Acute Physiology Score (SAPS) III [33] and the Sequential Organ Failure Assessment (SOFA) at admission [34].

Need of mechanical ventilation (MV), intensive care unit (ICU), hospital length of stay (LOS), and mortality rates from any cause were also assessed. All patients were followed up with until death or hospital discharge. One investigator conducted a revision of the data collected through a random check of 10% of all datasets in order to confirm internal validity and data consistency. After data collection, patients’ individual data with missing values per variable rate above 10% were excluded from the initial analysis. The remaining missing values were handled under the assumption of missing at random and no imputation methods were applied (missing values percentage negligibly small (1.257%)) [35].

CRP, procalcitonin (PCT), IL-6 and D-dimer levels, and leucocyte and lymphocyte counts were measured at ICU admission, considering their value as inflammatory biomarkers in COVID-19 patients [20,21,22,23,24,25,26]. Lymphocyte serum counts, regardless of the leucocyte serum counts analysis, were considered, due to the previously collected evidence of an independent association between disease severity and mortality in COVID-19-patients [17,18]. Biomarkers levels were determined with the Roche Cobas Integra 800 analyzer (Roche Diagnostic, Indianapolis, IN, USA).

The main outcome of interest was to correlate clinical and biomarker signatures at ICU admission to derive COVID-19 phenotypes and determine their correlation with clinical outcomes. Other measured outcomes were organ dysfunctions assessed by SOFA, ICU and hospital LOS, and mortality.

### 2.3. Data Processing and Statistical Analysis

Baseline clinical and serum data collected were considered for the Principal Component Analysis and clustering after data processing as previously described, confirming its completeness, consistency, and missing values.

Variables selected to be included for modeling were age, SAPS III score, SOFA score, CRP, D-dimer, PCT, IL-6 levels, and leucocyte and lymphocyte counts at ICU admission after standardization, in order to reduce magnitude effect.

We used agglomerative clustering on the selected variables using Ward’s method and applying squared Euclidean Distance as the similarity measure [36]. The classification was conducted without consideration of clinical outcomes. The aim of this statistical method was to find relatively homogeneous clusters of cases based on measured characteristics. The log-likelihood method was used to determine inter-subject distance and specific classification of participants. The model was produced using the Schwarz-Bayesian criterion, aiming to identify the most goodness of fit of the clustering model. We considered grouping patients into 2–6 phenotypes and determined the optimal number of three as the model with higher goodness of fit. The number of clusters was confirmed using an elbow plot, where the inflection point was used as a cutoff.

We then performed a Principal Component Analysis on the average covariance matrix to visualize the relationships among the three phenotypes and assess variable contributions.

Once the clusters were determined, we compared the association between classes and clinical outcomes (ICU and hospital LOS, mortality, MV need, vasopressor support, and renal replacement therapy) using logistic regression models. We used standard descriptive statistics and reported continuous variables as median [25–75% interquartile range]. Comparisons between groups were performed with two-tailed unpaired Student *t*-test, one-way ANOVA, Mann-Whitney U, or Kruskal-Wallis H tests for continuous variables according to data distribution. The Fisher exact test and Chi-square test were used to carry out comparisons between categorical variables as appropriate. All Gaussian distributed variables were expressed as mean (SD) and non-normally distributed variables as median (interquartile range [IQR]). Categorical variables were expressed as numbers and percentages.

We carried out all statistical analyses using the SPSS 23.0 software package (Chicago, IL, USA) and Prism 6.0 (Graphpad, Boston, MA, USA).

## 3. Results

### 3.1. Cluster Analysis

**Figure 1:**
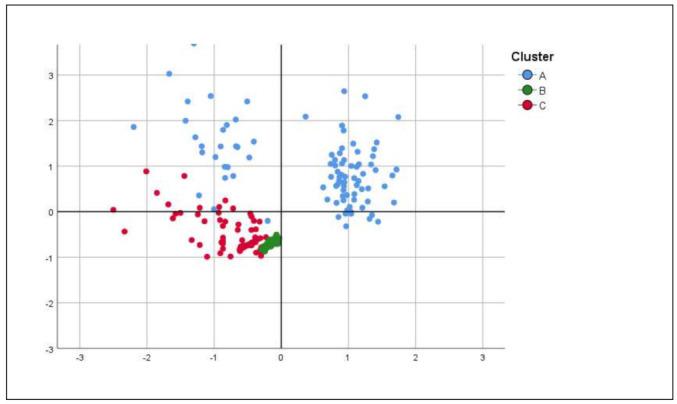
Factor map of the Principal Component Analysis showing the distribution of each patient in each cluster.

In total, 814 patients were included. The main demographics and clinical characteristics are depicted in Table 1. The cluster analysis revealed a three-class model as the most useful in the analyzed population, as presented in Table 2. The principal component analysis also defined three principal population-describing factors as appreciated through an elbow plot (Figure S1, Tables S1 and S2 of Supplementary Materials), supporting that three-class model after plotting for those factors (Figure 1).

This three-class model allocated 407 patients in phenotype A, 244 patients in phenotype B, and 163 patients in phenotype C, allowing for the definition of three distinct COVID-19 phenotypes (Table 1).

### 3.2. Phenotype’s Characterization and Clinical Outcomes

The main demographic, clinical, and analytical characteristics among the three phenotypes are presented in Table 1.

A clear distinction can be perceived in phenotype A versus phenotypes B and C. Phenotype A patients are significantly older, with a higher severity index (either by SOFA or SAPS III) and a higher requirement of organ support (either ventilatory or cardiovascular) (Table 1). Furthermore, these patients also showed significantly longer ICU and hospital LOS, and a higher in-hospital mortality rate.

Phenotypes B and C, on the other hand, demonstrate some overlapping of clinical and demographic characteristics but with markedly different outcomes. Although no difference is found between these two phenotypes regarding age, SOFA score, and SAPS III at ICU admission, and ventilatory, vasopressor, and renal supports, patients with phenotype C have higher ICU and hospital LOS (10 (3;10) days vs. 6 (5;11) days and 13 (2;14) days vs. 9 (1;14) days, respectively, *p* < 0.001) but a significantly lower in-hospital mortality rate (1.2% vs. 7%, respectively, *p* = 0.007), when compared to patients with phenotype B.

### 3.3. Phenotype’s Biomarker Profile

Higher baseline inflammatory markers were associated with a higher mortality rate (*p* = 0.001). Phenotype A patients presented with higher CRP, PCT, and IL-6 serum levels at ICU admission (*p* < 0.001). Furthermore, this phenotype also presented higher white blood cell counts, although no difference was registered in the lymphocyte count between groups.

Phenotypes B and C presented clearly different inflammatory profiles and mortality rates. Phenotype C patients presented higher PCT and IL-6 serum levels with lower CRP serum levels and mortality rate (1.2%). On the other hand, phenotype B patients presented lower PCT and IL-6 serum levels and higher CRP serum levels and a 4-fold higher mortality rate (7%).

## 4. Discussion

In our large cohort of severe COVID-19 patients, we were able to identify three distinct COVID-19 phenotypes. These phenotypes show significant differences in their clinical features, inflammatory profiles, and hospital outcomes. Our findings urge the necessity of comprehending COVID-19 as a disease that encompasses several patients with heterogenous outcomes. This may potentially prompt more patient-targeting therapeutic protocols and promote a medical practice based in more individualized and targeted approach to these patients.

Firstly, our cluster analysis identified a high-mortality rate phenotype (phenotype A) with an in-hospital mortality rate of 12%. This phenotype is clearly composed of older patients with high SOFA and SAPS III at ICU admission, a higher pro-inflammatory profile, and poor outcomes. This data reproduces previous evidence collected, describing a strong correlation between mortality rate and age, high CRP and PCT levels, and severity indexes at ICU admission in COVID-19 patients [37,38,39,40,41,42,43]. Clearly, this phenotype convenes the most severe COVID-19 patients, with significant differences to phenotypes B and C.

Phenotypes B and C, on the other hand, represent subpopulations of younger patients with lower severity scores and fewer organ support requirements. Albeit contrastingly different when comparing to phenotype A, these phenotypes B and C lose their apparent overlapping characteristics when their inflammatory biomarker profiles and mortality rates are considered. In fact, patients with phenotype C exhibit higher PCT and IL-6 serum levels with lower CRP serum levels and lower in-hospital mortality rates.

These results of COVID-19 patients’ phenotypes also demonstrate biological plausibility and are strikingly similar to those previously described by Azoulay et al. [6]. The authors reported the clinical and laboratory features of a cohort of 85 consecutive COVID-19 patients admitted to ICU. In this study, the hierarchical clustering also identified three clinical phenotypes of COVID-19 patients with a significant overlap with those identified in our study. Based on a bigger cohort of COVID-19 patients, our hierarchical clustering documents the same three phenotypes, with similar clinical profiles, inflammatory biomarker patterns, and hospital outcomes.

Our results are also consistently overlapping and complementary to those found in other COVID-19 phenotype-defining studies [10,11,12,13,14,15,16,27]. In a study by Lusczek et al., three clinical phenotypes were identified with similar clinical characteristics to those described in our study. A high-mortality rate phenotype composed of older patients with a higher number of comorbidities and higher in-hospital complications and CRP serum levels at admission was also described. Correspondingly, it also described two less-ill phenotypes of patients with lower mortality rates and lower inflammatory profiles [13]. The same results were also found in studies by Rodríguez et al. and Siepel et al., which deployed unsupervised clustering analysis of critically ill patients with COVID-19 in Spain and in the Netherlands, respectively [9,14]. The derived clinical phenotypes exhibit a marked similarity to our findings and significantly correlated with host-response patterns and ICU mortality. Across these studies, three phenotypes were identified with different high severity of illness and ICU mortality rates, and prominent dissimilarities between clinical phenotypes were associated to different inflammation profiles.

Although no statistical difference was found allowing for the definition of an inflammatory biomarker profile to segregate patient phenotypes B and C, this data is strikingly different from evidence previously collected [41,42]. Most of those results, suggesting a linear association between PCT, CRP, and IL-6 serum levels and patients’ mortality rate, stemmed from retrospective and observational analysis. Our results do not support a direct association between IL-6 and PCT serum levels and mortality rate in severe COVID-19 patients but suggest that CRP may be a more reliable surrogate of COVID-19 patient’s outcomes [44,45,46,47].

These findings could be somehow related to the heterogeneity of RCT results, namely of IL-6 receptor inhibitors [48]. Our and Azoulay’s results raised the possibility of a lack of effect or even harm of therapeutic options targeting this individual biomarker [7]. Pinpointing these isolated biomarkers as therapeutical targets may be insufficient to address all the inflammatory and mechanisms active in these patients. Furthermore, the prognostic value of this biomarker may be underlined in a more complex inflammatory profile, dependent on patient’s immunological variables, and subject to a different prevalence of these clinical phenotypes [49,50,51,52].

Our study conveys several strengths. It is composed of a large cohort of COVID-19 patients for a multinational and multicenter cluster analysis, circumventing some possible sources of bias. However, it is not without limitations. It is an observational study and, although it represents a large sample size of COVID-19 patients, it fails to document a clear segregation between the inflammatory profiles of the three clinical phenotypes that could reliably identify a subset of patients with higher mortality rates. A higher number of patients could also improve phenotype homogeneity. Furthermore, an external validation of the clusters is absent, undermining the potential of finding these phenotypes in other studied COVID-19 populations. Moreover, it convenes a study period of two variants and significant therapeutic recommendation changes, specifically, but not limited to, in the use of invasive and non-invasive mechanical ventilation, that could count for differences within the studied population. Furthermore, we recognize a significant absence of data concerning time from onset of symptoms and time until intubation and mechanical ventilation. Finally, COVID-19 treatment effects were not evaluated, and long-term follow-up of patients was not performed. Therefore, the impact of these phenotypes on their long-term mortality rate was not evaluated.

## 5. Conclusions

Severe COVID-19 patients exhibit three different phenotypes with distinct clinical and biochemical profiles and outcomes. Our data presents a clearly high-mortality group with a high level of organ requirements that should be promptly recognized in order to improve survivability and early organ support. It also suggests an association of CRP serum levels with patients’ outcomes. In contrast to our expectations, there was no evidence of any association between IL-6 serum levels and patients’ outcomes, namely in phenotype C, which presented intermediate levels of Il-6 serum levels and low patient mortality. Further validation of these phenotypes with external cohorts could allow the implementation of point-of-care biomarker determinations to guide patients’ management.

Our data also conveys that different patients’ profiles may translate to an early identification of patients with variable responses to certain COVID-19 directed therapies and may help understand heterogeneity of treatment effects. It highlights different prognoses in similar-appearing patients with COVID-19, reinforcing the potential use of biochemical phenotyping in the clinical characterization of these patients. Furthermore, it may provide impactful insights in future randomized clinical trial analyses and a concept for trial enrichment.

## Figures and Tables

**Table 1:** Demographic, clinical and outcome variables of patients according to the COVID-19 phenotype *.

	PHENOTYPE A (*n* = 407; 60%)	PHENOTYPE B (*n* = 244; 24%)	PHENOTYPE C (*n* = 163; 16%)	*p* ¥
Age, years, (median (IQR))	81 (65–97)	62 (47–79)	63 (47–80)	<0.001
Gender, males, (*n*, %)	221 (54.3%)	131 (53.7%)	78 (47.9%)	0.08
**Previous Medical Comorbidities**				
Chronic Obstructive Pulmonary Disease (*n*, %)	37 (9.1%)	18 (7.4%)	14 (8.9%)	0.528
Asthma (*n*, %)	18 (4.4%)	8 (3.3%)	5 (3.1%)	0.347
Chronic Kidney Disease (*n*, %)	89 (21.9%)	29 (11.9%)	23 (14.1%)	0.125
Obesity (*n*, %)	64 (15.7%)	36 (14.8%)	27 (16.7%)	0.214
Diabetes Mellitus (*n*, %)	129 (31.7%)	82 (33.6%)	47 (28.8%)	0.08
Ischemic Cardiopathy (*n*, %)	147 (36.1%)	64 (26.2%)	39 (23.9%)	0.04
SOFA at admission (median (IQR))	10 (5; 13)	3 (2; 5)	1 (0; 3)	<0.001
SAPS III at admission (mean ± SD)	78 ± 10	50 ± 7	47 ± 12	<0.001
Mechanical Ventilation (*n*, %)	173 (42.5%)	11 (4.6%)	11 (6.9%)	<0.001
Vasopressor Support (*n*, %)	112 (27.5%)	38 (15.6%)	38 (14.4%)	<0.001
Renal replacement therapy (*n*, %)	58 (14.2%)	19 (7.8%)	19 (11.6%)	0.152
**Laboratory results**				
C reactive Protein at admission, mg/dL (median (IQR))	32.3 (24.8; 81.5)	20.0 (10.3; 40.6)	17.20 (4.0; 23.7)	0.013
Max registered C-Reactive protein, mg/dL (mean ± SD)	32.3 ± 11.0	25.3 ± 10.4	18.6 ± 12.5	<0.001
Procalcitonin at admission, ng/mL (median (IQR))	3.30 (0.55; 3.35)	0.17 (0.05; 0.23)	0.22 (0.12; 0.23)	<0.001
Max registered Procalcitonin, ng/mL (median (IQR))	9.73 (0.86; 13.54)	0.34 (0.06; 0.74)	1.30 (0.70; 1.40)	<0.001
D-dimer level at admission, ng/mL (median (IQR))	1165 (587; 1663)	610 (97; 753)	202 (119; 262)	0.003
Max D-dimer registered, ng/mL (median (IQR)	2778 (875; 3822)	655 (48; 1305)	303 (78; 307)	0.018
Minimum Leucocyte count registered, ×10^9^ (mean ± SD)	11.0 ± 7.09	5.0 ± 2.03	5.3 ± 2.2	<0.001
Minimum Lymphocyte count registered, ×10^9^ (median (IQR))	0.52 (0.32; 0.62)	0.36 (0.12; 0.39)	0.57 (0.68; 1.09)	0.146
IL-6 serum levels, mg/mL (median (IQR))	57.9 (5.7; 61.0)	35.4 (6.6; 42.7)	41.0 (16.0; 49.0)	0.01
Remdesivir, (*n*, %)	359 (88.2%)	243 (99.6%)	147 (90.2%)	0.167
Corticosteroid therapy (*n*, %)	176 (43.2%)	137 (97.9%)	147 (90.0%)	0.001
Ventilator-free days, days, (median (IQR))	23 (20; 24)	12 (4; 20)	10 (4; 17)	0.001
ICU length of stay, days, (median (IQR))	14 (11; 15)	6 (5; 11)	10 (3; 10)	<0.001
Hospital Length of stay, days, (mean ± sd)	18 (7; 20)	9 (1; 14)	13 (2; 14)	0.001

* IQR denotes Interquartile range and SD denotes standard deviation. ¥ *p*-values were determined using Chi-square test, ANOVA test and Kruskal-Wallis H test.

**Table 2:** Cluster class fitting analysis.

Number of Individuals per Class/Phenotype							
Number of Classes	BIC ¥	Entropy *	N1	N2	N3	N4	*p*-Value **
2	6034.4	0.77	787	27			0.227
3	2094.1	0.87	244	163	407		0.007
4	8056.7	0.52	11	44	757	6	0.183

¥ BIC stands for Bayesian information criterion. * Entropy is an index of how well the classes are separated. It ranges from zero to one and values around 0.8 and up are generally considered a sign of a useful model. ** By likelihood ratio test, testing whether the number of classes improved the model fitness compared to a model using one fewer class.

## Data Availability

The datasets generated and/or analyzed during the current study are not publicly available due to privacy issues but are available from the corresponding author on reasonable request.

## Supplementary Materials

The following supporting information can be downloaded at: https://www.mdpi.com/article/10.3390/jcm12083035/s1, Figure S1: Principal Component analysis using elbow method of the cluster dataset; Table S1: Principal Components analysis (PCA) of the cluster dataset; Table S2: Demographic, clinical and outcome variables of patients between Phenotypes B and C.
