# Supplementary material for: Identification of Distinct Clinical Phenotypes of Critically Ill COVID-19 Patients: Results from a Cohort Observational Study
Source: J Clin Med. 2023 Apr 21;12(8):3035. doi: 10.3390/jcm12083035 (PMC10144996; doi:10.3390/jcm12083035)
Supplement: Supplementary file 1 [file jcm-12-03035-s001.zip › jcm-2303861-supplementary.pdf]

Supplemmentary Material

Title: Identification of Distinct Clinical Phenotypes of Critically Ill COVID-19 Patients: Results from a Cohort Observational Study

José Pedro Cidade, Vicente Cés de Souza Dantas, Alessandra de Figueiredo Thompson, Renata Carnevale Carneiro Chermont de Miranda, Rafaela Mamfrim, Henrique Caroli, Gabriela Escudini, Natalia Oliveira, Taiza Castro, Renata Oliveira, Pedro Póvoa

Table S1– Principal Components analysis (PCA) of the cluster dataset

Explained total variance						
Component	Initial eigenvalues			Extraction Sums of Squared Loadings		
	Total	% of variance	% cumulative	Total	% of variance	% cumulative
C-reactive Protein serum level	1,602	53,416	53,416	1,602	53,416	53,416
Procalcitonin serum level	1,010	33,677	87,092	1,010	33,677	87,092
Il-6 serum level	1,005	9,908	97,000	1,005	9,908	97,000
Leukocyte count	,561	1,810	98,81			
D-dimer serum level	,471	,908	99,72			
Lymphocyte count	,387	,028	100,000			

Note: Kaiser-Meyer-Olkin measure: 0.895

Table S2– Demographic, clinical and outcome variables of patients between Phenotypes B and C*

	PHENOTYPE B (n=244; 24%)	PHENOTYPE C (n=163; 16%)	p¥
Age, years, (median (IQR))	62 (47-79)	63 (47-80)	0.9

Gender, males, (n, %)	131 (53.7%)	78 (47.9%)	0.08
**Previous Medical Comorbidities**			
Chronic Obstructive Pulmonary Disease (n ,%)	18 (7.4%)	14 (8.9%)	0.09
Asthma (n, %)	8 (3.3%)	5 (3.1%)	0.45
Chronic Kidney Disease (n, %)	29 (11.9%)	23 (14.1%)	0.127
Obesity (n, %)	36 (14.8%)	27 (16.7%)	0.298
Diabetes Mellitus (n, %)	82 (33.6%)	47 (28.8%)	0.12
Ischemic Cardiopathy (n, %)	64 (26.2%)	39 (23.9%)	0.659
SOFA at admission (median (IQR))	3 (2;5)	1 (0; 3)	**0.04**
SAPS III at admission (mean ± SD)	50 ± 7	47 ± 12	0.413
Mechanical Ventilation (n, %)	11 (4.6%)	11 (6.9%)	0.06
Vasopressor Support (n, %)	38 (15.6%)	38 (14.4%)	0.318
Renal replacement therapy (n, %)	19 (7.8%)	19 (11.6%)	0.59
**Laboratory results**			
C reactive Protein at admission, mg/dL (median (IQR))	20.0 (10.3; 40.6)	17.20 (4.0; 23.7)	**0.001**
Max registered C-Reactive protein, mg/dL (mean ± SD)	25.3 ± 10.4	18.6 ± 12.5	**<0.001**
Procalcitonin at admission, ng/mL (median (IQR))	0.17 (0.05; 0.23)	0.22 (0.12; 0.23)	**0.02**
Max registered Procalcitonin, ng/mL (median (IQR))	0.34 (0.06; 0.74)	1.30 (0.70; 1.40)	**0.035**
D-dimer level at admission, ng/mL (median (IQR))	610 (97; 753)	202 (119; 262)	**0.001**
Max D-dimer registered, ng/mL (median (IQR)	655 (48; 1305)	303 (78; 307)	**0.001**
Minimum Leucocyte count registered, $x10^9$ (mean ± SD)	5.0 ± 2.03	5.3 ± 2.2	**<0.001**
Minimum Lymphocyte count registered, $x10^9$ (median (IQR))	0.36 (0.12; 0.39)	0.57 (0.68; 1.09)	0.745
IL-6 serum levels, mg/mL (median (IQR))	35.4 (6.6; 42.7)	41.0 (16.0; 49.0)	**0.001**
Remdesivir, (n, %)	243 (99.6%)	147 (90.2%)	**0.03**
Corticosteroid therapy (n, %)	137 (97.9%)	147 (90.0%)	**0.001**
Ventilator-free days, days, (median (IQR))	12 (4; 20)	10 (4;17)	0.568
ICU length of stay, days, (median (IQR))	6 (5; 11)	10 (3; 10)	**0.01**
Hospital Length of stay, days, (mean ± sd)	9 (1; 14)	13 (2; 14)	**0.001**
Mortality, (n, %)	17 (7.0%)	2 (1.2%)	**<0.001**

* IQR denotes Interquartile range and SD denotes standard deviation

¥ p-values were determined using Chi-square test, ANOVA test and Kruskal-Wallis H test

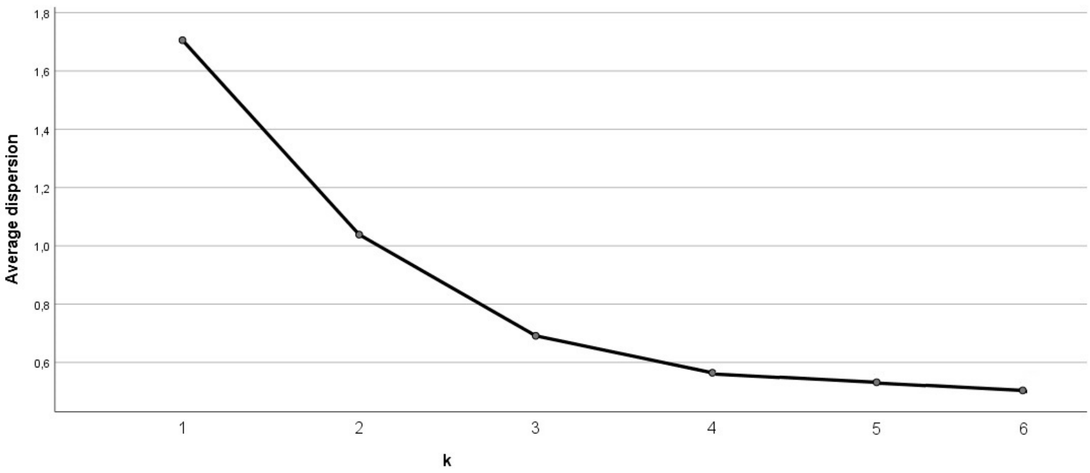

Figure S1– Principal Component analysis using elbow method of the cluster dataset
